# Functional NIRS Measurement of Cytochrome-C-Oxidase Demonstrates a More Brain-Specific Marker of Frontal Lobe Activation Compared to the Haemoglobins

**DOI:** 10.1007/978-3-319-55231-6_19

**Published:** 2017-03-21

**Authors:** Isabel de Roever, Gemma Bale, Robert J. Cooper, Ilias Tachtsidis

**Affiliations:** Department of Medical Physics and Bioengineering, University College London, London, UK

**Keywords:** Near-infrared spectroscopy, Functional activation, Cytochrome-c-oxidase, Haemodynamics, Short-separation regression

## Abstract

Functional near-infrared spectroscopy (fNIRS) is an increasingly common neuromonitoring technique used to observe evoked haemodynamic changes in the brain in response to a stimulus. The measurement is typically in terms of concentration changes of oxy- (∆HbO_2_) and deoxy- (∆HHb) haemoglobin. However, noise from systemic fluctuations in the concentration of these chromophores can contaminate stimulus-evoked haemodynamic responses, leading to misinterpretation of results. Short-separation channels can be used to regress out extracerebral haemodynamics to better reveal cerebral changes, significantly improving the reliability of fNIRS. Broadband NIRS can be used to additionally monitor concentration changes of the oxidation state of cytochrome-c-oxidase (∆oxCCO). Recent studies have shown ∆oxCCO to be a depth-dependent and hence brain-specific signal. This study aims to investigate whether ∆oxCCO can produce a more robust marker of functional activation. Continuous frontal lobe NIRS measurements were collected from 17 healthy adult volunteers. Short 1 cm source-detector separation channels were regressed from longer separation channels in order to minimise the extracerebral contribution to standard fNIRS channels. Significant changes in ∆HbO_2_ and ∆HHb were seen at 1 cm channels but were not observed in ∆oxCCO. An improvement in the haemodynamic signals was achieved with regression of the 1 cm channel. Broadband NIRS-measured concentration changes of the oxidation state of cytochrome-c-oxidase has the potential to be an alternative and more brain-specific marker of functional activation.

## Introduction

Functional near-infrared spectroscopy is commonly used to monitor stimulus-evoked cerebral haemodynamic responses due to neurovascular coupling, via measured regional concentration changes of Functional near-infrared spectroscopy (fNIRS). A typical functional activation haemodynamic response function consists of an increase in ∆HbO_2_ and a concurrent, co-located decrease in ∆HHb. However, one of the main problems with fNIRS is its susceptibility to noise. This can arise from two main Functional near-infrared spectroscopy (fNIRS): task-related systemic activity and systemic-driven changes in the extracerebral layers [1]. The latter is a significant source of interference due to the nature of fNIRS Functional near-infrared spectroscopy (fNIRS), where light propagates through the superficial layers upon emission and detection in reflectance mode, and hence is highly sensitive to changes in these superficial layers [2].

An increasingly common method to reduce contributions from extracerebral layers is with the use of Functional near-infrared spectroscopy (fNIRS) NIRS channels to sample only those layers, and use of the resulting signals as regressors in a linear model of the longer fNIRS channels [3]. It has been shown that a short source-detector separation of ~0.8 cm can effectively sample the extracerebral layer [2]. Hence, short channels can facilitate removal of superficial contamination from standard fNIRS channels, significantly improving the reliability of fNIRS measurements.

Using broadband NIRS we can additionally monitor oxidation changes of Cytochrome-c-oxidase (CCO). Functional near-infrared spectroscopy (fNIRS) is the terminal electron acceptor in the electron transport chain in the mitochondria and is responsible for more than 95% of oxygen metabolism. Measurements of ∆oxCCO therefore provides information about oxygen utilisation at a cellular level [4]. The concentration of CCO is less than 10% in vivo than that of haemoglobin, hence a broadband NIRS system is required to accurately separate changes in attenuation due to this chromophore [5].

Recently, it has been shown using a Broadband NIRS system with multiple source-detector separations that ∆oxCCO signals display a depth-dependence not seen in haemodynamic signals during oxygen delivery challenges in the healthy adult head [6]. The ∆oxCCO response was only seen at longer source-detector separations (3.5 cm) compared to ∆HbO_2_ and ∆HHb, which showed a response at all source-detector separations (2, 2.5, 3 and 3.5 cm) interrogating both scalp and brain. This depth-dependence suggests CCO is brain-specific; this is likely due to the higher mitochondrial density in tissue with higher metabolic rates such as the brain compared to tissue with lower metabolic rates [6]. The ∆oxCCO signal therefore offers an alternative cerebral optical signal to haemodynamic signals.

This study aims to investigate the use of ∆oxCCO as an alternative and more robust marker of functional activation due to its brain-specificity compared to haemoglobin signals. The extracerebral contribution to the Functional near-infrared spectroscopy (fNIRS) is investigated with the use of a multi-distance broadband NIRS system, with short separation of 1 cm to probe mostly the skin, scalp and skull.

## Functional near-infrared spectroscopy (fNIRS)

Healthy adult volunteers were recruited for this study and written informed consent obtained. The study was approved by the University College London (UCL) Ethics Committee. Data were collected from 17 healthy adult volunteers (13 male; age range 22–34 years) during functional activation. A working memory challenge in the form of either a Stroop task or anagram-solving task was used in order to induce a frontal haemodynamic response. Data were collected continuously for a 30 s baseline period, followed by 30 s of activation and 30 s of rest, alternated 4 times. The total acquisition time was therefore 270 s.

**Fig. 19.1:**
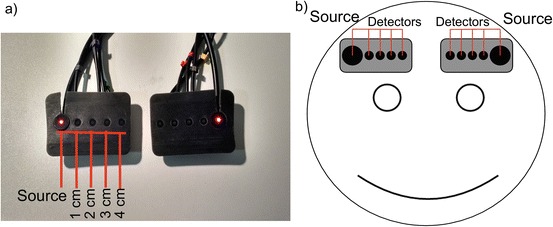
(**a**) Probe holdersFunctional near-infrared spectroscopy (fNIRS) with source and 4 detectors. (**b**) Diagram showing probe placement

Data were collected using a broadband NIRS system called CYRIL (CYtochrome Research Instrument and appLication)CYtochrome Research Instrument and appLication (CYRIL), previously described [7]. It is an 8-channel system with two light emission fibres; optode holders were placed on the right and left sides of the forehead of each subject as shown in Fig. 19.1. Intensity data from the 4 cm channels displayed poor signal-to-noise ratio so were excluded from further analysis.

CYRIL is comprised of a white light source and a lens-based spectrometer with the capacity to collect light intensity data for 136 wavelengths between 771–906 nm at 1 Hz sampling rate. Measured changes in light attenuation were then converted to concentration changes of ∆HbO_2_, ∆HHb and ∆oxCCO using the UCLn algorithm [5]. As the differential pathlength factor is likely to vary across different source-detector separations, no pathlength was used. Instead, concentration data are presented in terms of μM.cm.

Data analysis was carried out in MATLAB (Mathworks, USA) and using modified versions of functions from the HOMER2 NIRS processing package (http://www.homer-fnirs.org). Concentration data were zero-meaned and bandpass-filtered in the range [0.005 0.3] Hz, using a 5th order Butterworth filter, to remove physiological noise. The haemodynamic response was then extracted using one of two methods: the first was a block-average across all the events for all the subjects to isolate stimulus-related activations from uncorrelated haemodynamic trends (‘Block average method’); the second involved regression of the 1 cm channel by building a simple general linear model of each chromophore signal from the 2 and 3 cm channels (‘Short-separation regression method’). This general linear model uses a series of Gaussian basis functions convolved with a vector defining the time of the stimulus presentation to model the haemodynamic response function [8].

All statistical analysis was carried out in SPSS (IBM, USA). A baseline period was defined as the 10 s window immediately prior to the onset of activation, and 10 s activation period was chosen around the maximum change in the middle of the stimulus period. The response for each subject was defined as the difference between activation and baselineFunctional near-infrared spectroscopy (fNIRS). Student’s unpaired, one-sided t-test was used to compare the response of the activation compared to zero (i.e. no response) and values of p < 0.05 were considered significant. Results are presented as mean ± standard error.

## Results

**Fig. 19.2:**
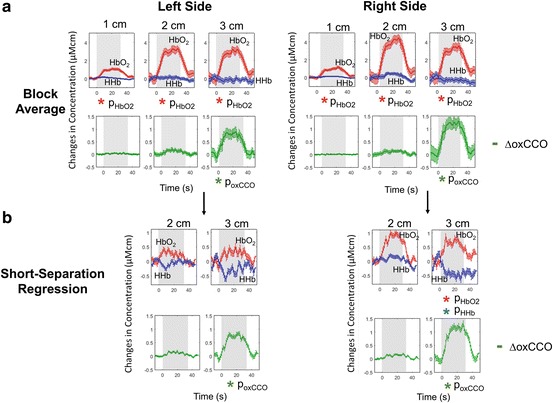
Functional near-infrared spectroscopy (fNIRS) of mean for 17 subjects during functional activation for left and right sides (**a**) without regression (**b**) with short-separation regression. Stimulus period indicated by *grey* background

The block average and short-separation regressed results for the 1, 2 and 3 cm channels are shown in Fig. 19.2.

Results of the Functional near-infrared spectroscopy (fNIRS) for the right side show an increase in ∆HbO_2_ at all source-detector separations, with a mean increase of 1.2 ± 0.5 μM.cm at 1 cm, 4.5 ± 1.6 μM.cm at 2 cm and 3.6 ± 1.6 μM.cm at 3 cm. No response is seen in ∆HHb at any of the channels. The ∆oxCCO shows no significant response at 1 cm and 2 cm but a large response is seen at 3 cm of 1.3 ± 0.8 μM.cm. Results for the Short-separation regression method show a significant increase in ∆HbO_2_ at only 3 cm of 0.9 ± 0.4 μM.cm, whilst ∆HHb now displays a significant decrease at 3 cm of −0.6 ± 0.5 μM.cm. The ∆oxCCO shows similar results as before, with the mean response of 1.3 ± 0.4 μM.cm at 3 cm.

Results for the block-average method for the left side show an increase in ∆HbO_2_ at all separations, with mean values of 1.1 ± 0.4 μM.cm at 1 cm, 3.3 ± 1.3 μM.cm at 2 cm and 3.2 ± 1.5 μM.cm at 3 cm. There is no significant change in ∆HHb at any of the separations. ∆oxCCO shows a significant increase at only the 3 cm channel of 0.9 ± 0.8 μM.cm. After short-separation regression, no significant changes are seen in either of the haemodynamic variables. Functional near-infrared spectroscopy (fNIRS) displays a similar response as to the block average, with a significant increase of 0.8 ± 0.3 μM.cm at 3 cm.

## Discussion

We observed a response in ∆oxCCO in only the longer source-detector separation of 3 cm but not in the shorter separations of 1 cm and 2 cm. The regression of the 1 cm channel made no difference to the Functional near-infrared spectroscopy (fNIRS) response.

There was a response in ∆HbO_2_ Functional near-infrared spectroscopy (fNIRS) at all source-detector separations, whereas ∆HHb showed no significant changes in any of the channels. After short-separation regression, the 2 cm channel did not show any significant response in the ∆HbO_2_ and ∆HHb signals for either side, whilst at 3 cm there was a significant increase in ∆HbO_2_ and decrease in ∆HHb for the right side and a similar but not significant trend was seen for the left side.

A scalp response was seen in both the right and left sides in the 1 cm channel. Results for the right side block-average at 3 cm show a false negative; no functional activation observed from the Functional near-infrared spectroscopy (fNIRS) due to the absence of a significant decrease in ∆HHb. However, ∆oxCCO shows a strong increase that is indicative of functional activation. When the 1 cm short separation is regressed from the longer channels, we see a functional response in the haemoglobins not previously seen, with a significant increase in ∆HbO_2_ and now significant decrease in ∆HHb. If only the changes in ∆HbO_2_ were reported, then at 1 cm and 2 cm one would observe functional activation; however, taking into account ∆oxCCO and the short-separation regressed ∆HHb signals, we observe no functional activation in these channels. In this case, the ∆oxCCO and regressed ∆HHb signals have the capacity to rule out false positives.

Results for the left hemisphere show no functional activation from the haemoglobin signals at 3 cm even after regression. If we rely on the significant increase in ∆oxCCO as an indicator of Functional near-infrared spectroscopy (fNIRS), the haemodynamic result is a false negative. The 1 cm short-separation regression in this case did not have the capacity to recover the false negative in the haemodynamic signals, which may be due to the scalp response not being fully removed. A false negative could also be caused by systemic changes such as blood pressure producing a significant effect on the Functional near-infrared spectroscopy (fNIRS) that masked activation. We did not measure systemic variables so cannot comment on the cause. However, previous studies have found systemic task-related changes to influence NIRS signals [9].

Note that the amplitude of the haemoglobin signals is on a similar scale to that of the ∆oxCCO signals after regression. This could be due to the fundamental characteristics of the regression method. The Least-squares static estimator regression approach employed here can result in an underestimation of the haemodynamic response: either because the short-separation measurement has a non-negligible sensitivity to cortical tissue or because the superficial short-separation signal during the functional response mimics the true cerebral response. This effect could likely be improved through the use of a dynamic estimation approach, such as the Kalman filter methodology employed by Gagnon et al. [10].

This study demonstrates that inclusion of the ∆oxCCO signal during functional activation studies with fNIRS can provide an additional robust marker of brain activation allowing better identification of false positives and negatives.
